# Mobile health-technology integrated care in secondary prevention atrial fibrillation patients: a post-hoc analysis from the mAFA-II randomized clinical trial

**DOI:** 10.1007/s11739-023-03249-0

**Published:** 2023-03-16

**Authors:** Yutao Guo, Giulio Francesco Romiti, Dimitrios Sagris, Marco Proietti, Niccolò Bonini, Hui Zhang, Gregory Y. H. Lip, The mAF-App II trial investigators

**Affiliations:** 1 Department of Pulmonary Vessel and Thrombotic Disease, Sixth Medical Center, Chinese PLA General Hospital, Beijing, People’s Republic of China; 2 Liverpool Centre for Cardiovascular Sciences, University of Liverpool, Liverpool John Moores University and Liverpool Heart and Chest Hospital, William Henry Duncan Building, 6 West Derby St, Liverpool, L7 8TX UK; 3 Department of Translational and Precision Medicine, Sapienza – University of Rome, Rome, Italy; 4 Department of Internal Medicine, School of Health Sciences, Faculty of Medicine, University of Thessaly, Larissa, Greece; 5 Department of Clinical Sciences and Community Health, University of Milan, Milan, Italy; 6 Geriatric Unit, IRCCS Istituti Clinici Scientifici Maugeri, Milan, Italy; 7 Cardiology Division, Department of Biomedical, Metabolic and Neural Sciences, University of Modena and Reggio Emilia, PoliclinicoDi Modena, Modena, Italy; 8 Department of Clinical Medicine, Aalborg University, Aalborg, Denmark

**Keywords:** Atrial fibrillation, Integrated care, Stroke, Thromboembolism, Outcomes

## Abstract

AF patients with history of thromboembolic events are at higher risk of thromboembolic recurrences, despite appropriate antithrombotic treatment. We aimed to evaluate the effect of mobile health (mHealth) technology-implemented ‘Atrial fibrillation Better Care’ (ABC) pathway approach (mAFA intervention) in secondary prevention AF patients. The Mobile Health Technology for Improved Screening and Optimized Integrated Care in AF (mAFA-II) cluster randomized trial enrolled adult AF patients across 40 centers in China. The main outcome was the composite outcome of stroke or thromboembolism, all-cause death, and rehospitalization. Using Inverse Probability of Treatment Weighting (IPTW), we evaluated the effect of the mAFA intervention in patients with and without prior history of thromboembolic events (i.e., ischemic stroke or thromboembolism). Among the 3324 patients enrolled in the trial, 496 (14.9%, mean age: 75.1 ± 11.4 years, 35.9% females) had a previous episode of thromboembolic event. No significant interaction was observed for the effect of mAFA intervention in patients with vs. without history of thromboembolic events [Hazard ratio, (HR): 0.38, 95% confidence interval (CI):0.18–0.80 vs. HR 0.55, 95% CI 0.17–1.76, *p* for interaction = 0.587); however, a trend towards lower efficacy of mAFA intervention among AF patients in secondary prevention was observed for secondary outcomes, with significant interaction for bleeding events (*p* = 0.034) and the composite of cardiovascular events (*p* = 0.015). A mHealth-technology-implemented ABC pathway provided generally consistent reduction of the risk of primary outcome in both primary and secondary prevention AF patients. Secondary prevention patients may require further specific approaches to improve clinical outcomes such as bleeding and cardiovascular events.

Trial registration: WHO International Clinical Trials Registry Platform (ICTRP) Registration number ChiCTR-OOC-17014138.

## Introduction

Atrial Fibrillation (AF) is the most common arrhythmia worldwide, with increasing incidence and prevalence worldwide [1, 2]. Patients with AF are at higher risk of thromboembolic events [3]; oral anticoagulation (OAC) significantly reduces this risk, although not eliminating it, particularly in patients with previous thromboembolic events, who are at higher risk of cardiovascular events, death and recurrent ischemic events [4–7]. Indeed, international guidelines recommend OACs for the secondary prevention of thromboembolism in AF patients with history of stroke, transient ischemic attack (TIA) or thromboembolism [8–10]. Although the introduction of non-vitamin K antagonist oral anticoagulants (NOACs) have provided a safer and effective alternative to Vitamin K antagonist (VKA) for the secondary prevention of these patients [11], the risk of recurrent thromboembolic events is still considerable in this group of patient [6, 7], which therefore require further interventions, beyond appropriate antithrombotic treatment, to tackle the increased risk of thromboembolism.

Recent guidelines have advocated for the adoption of integrated care management approach to improve outcomes in AF patients [8, 10]. The ‘Atrial fibrillation Better Care’ (ABC) pathway has been indeed proposed to implement an integrated care approach in AF patients [12], based on three main pillars: A, anticoagulation/avoiding stroke; B, better symptom control; and C, cardiovascular risk and comorbidity optimization. In the Mobile Health Technology for Improved Screening and Optimized Integrated Care in AF (mAFA-II) prospective cluster randomized trial, a mobile health (mHealth) implemented ABC pathway (mAFA intervention) resulted in a significant reduction of the risk of the composite outcome of ischemic stroke/systemic thromboembolism, death, and hospitalization, compared to usual care in AF patients [13]. Consistently, a recent consensus statement from the European Society of Cardiology Council on Stroke postulated the need for a holistic approach to post-stroke care based on a modified ABC pathway [14]. Therefore, more evidence on the efficacy of an ABC-pathway adherent approach for the secondary prevention of thromboembolism in AF patients is needed.

In this post-hoc ancillary analysis of the mAFA-II trial, we aimed to evaluate the effect of the mAFA intervention in patients with history of previous ischemic stroke or systemic embolism, compared to those without.

## Methods

Details on the design and primary results of the mAFA-II trial have been previously published. [13, 15] Briefly, the mAFA-II trial recruited adult patients (≥ 18 years) with AF between June 2018 and August 2019, across 40 participating centers in China; centers were randomized in a 1:1 ratio to the mAFA intervention or usual care. All patients enrolled provided written informed consent. Main exclusion criteria were: subjects unable to provide informed consent, patients with moderate to severe mitral stenosis, or with mechanical prosthetic valve, and subjects unable to be followed up for 1 year for any reason. The trial was conducted according to the Consolidated Standards of Reporting Trials (CONSORT) reporting guideline and according to the Declaration of Helsinki, and was approved by the Central Medical Ethic Committee of Chinese PLA General Hospital and by local institutional review boards.

### ABC pathway implementation

Centres randomized to the mAFA intervention implemented the ABC pathway defined as follows:

- ‘A’ criterion: OAC treatment according to regular and dynamic re-assessment of thromboembolic and bleeding risks, with dose adjustments based on regular evaluation of renal and liver function;
- ‘B’ criterion: regular assessment of patient-reported symptoms, evaluated according to the European Heart Rhythm Association classification, and treated according to symptoms-directed management (including rhythm control therapies);
- ‘C’ criterion: management and optimization of concurrent comorbidities (e.g. hypertension management according to blood pressure monitoring, etc.).

Patients allocated to “usual care” group were managed according to local practices.

### Outcomes and follow-up

The incidence of clinical adverse events was assessed through 6 and 12-month follow-up of each patient. Consistently with the primary analysis of the trial, we defined the primary outcome for this analysis as the composite all-cause death, ischemic stroke or systemic thromboembolism, and rehospitalization. We also investigated several exploratory secondary outcomes: all-cause death, thromboembolism (i.e., ischemic stroke or systemic thromboembolism), bleeding events (intracranial and extracranial), the composite of cardiovascular outcomes (recurrent AF, heart failure and acute coronary syndrome), and rehospitalization. The effect of the mAFA intervention was evaluated, for each outcome, according to the history of previous thromboembolism (i.e., patients with vs. without ischemic stroke or systemic embolism).

### Statistical analysis

We reported continuous variables as mean and standard deviation (SD) if normally distributed or median and interquartile range (IQR) if non-normally distributed; categorical variables were reported as frequencies and percentage.

To ensure balance of baseline characteristics across subjects with and without history of thromboembolism, and according to the allocation to mAFA intervention or usual care, we calculated a subgroup-balancing propensity score (PS) [16] of receiving mAFA intervention, through a multivariable logistic regression model, which included 26 variables (age, sex, smoking status, type of AF, previous AF treatment, hypertension, diabetes, coronary artery disease (CAD), heart failure (HF), peripheral artery disease (PAD), renal and liver dysfunction, pulmonary hypertension, previous episodes of intracerebral hemorrhage or other bleeding, anemia, hyperthyroidism, cardiomyopathies (dilated or hypertrophic), and clinical risk scores, i.e. CHA_2_DS_2_-VASc and HAS-BLED). We then calculated the inverse probability of treatment weights (IPTW) according to the PS. Covariate balance after IPTW was assessed for continuous variables using standardized mean differences (SMD), and for binary variables using raw differences in proportion. Differences < 0.10 indicated adequate balance. Finally, we performed Cox regression models using IPTW and with robust estimation of SE to evaluate the interaction between history of thromboembolism and effect of mAFA intervention.

A 2-sided *p*-value < 0.05 was considered statistically significant. All statistical analyses were conducted using R 4.2.1 (R Foundation for Statistical Computing 2020, Vienna, Austria).

## Results

**Fig. 1:**
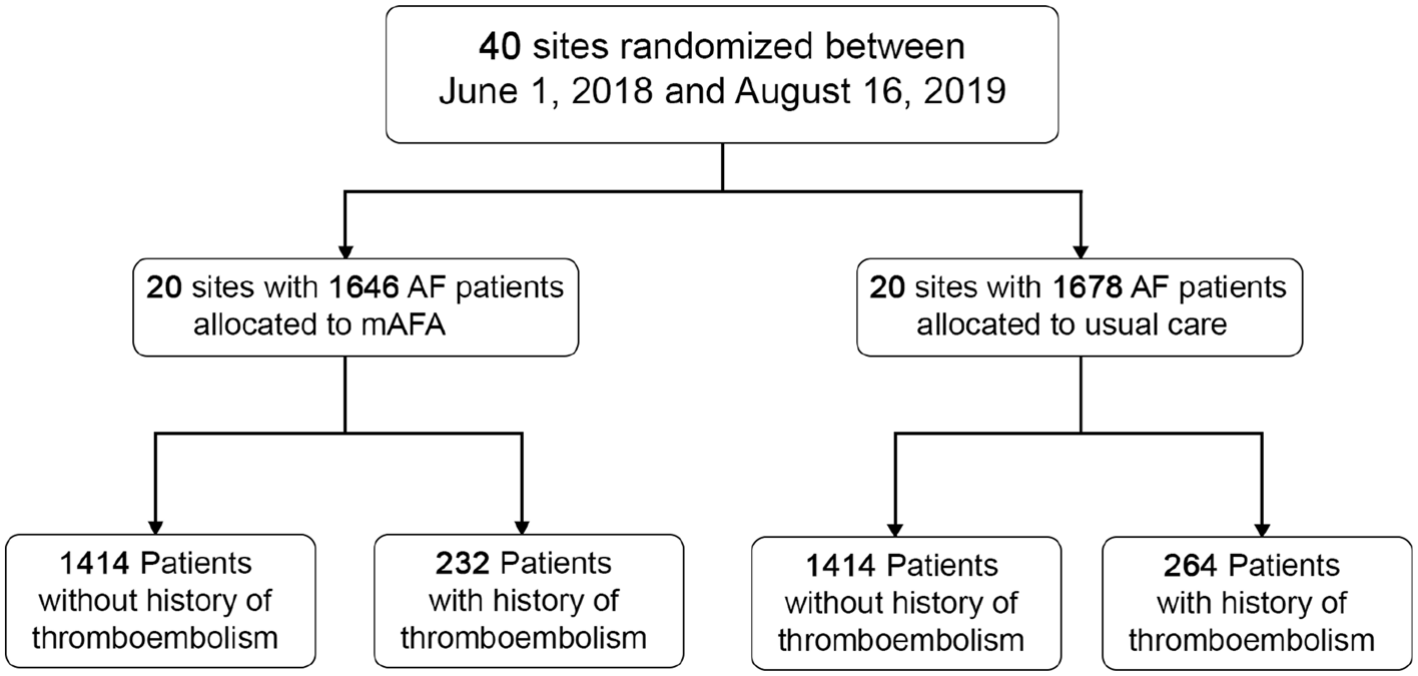
Study flow diagram

3324 patients were enrolled in the mAFA-II trial: 1646 subjects were allocated to the mAFA intervention, and 1678 were allocated to usual care (Fig. 1). At baseline, 496 (14.9%; mean age: 75.1 ± 11.4 years, 35.9% women) patients had a previous episode of thromboembolic event; these subjects were older, and with higher prevalences of comorbidities and cardiovascular risk factors (Supplemental Table 1).

### Patients with previous thromboembolic event

Baseline characteristics according to the history of previous thromboembolism and according to mAFA allocation are reported in Table 1, while treatments are reported in Supplemental Table 2. 232 (46.8%) patients with previous thromboembolism were allocated to the mAFA intervention, with a median follow-up of 257 (IQR 98–367) days, while 264 (53.2%) subjects with previous thromboembolic events were allocated to usual care, with a median follow-up of 287 (IQR 124–395) days. Women were more represented among the mAFA intervention group (50.0% vs. 23.5%, *p* < 0.001); moreover, patients allocated to mAFA intervention had higher prevalences of history of HF (57.8% vs. 20.1%, *p* < 0.001) and higher CHA_2_DS_2_-VASc score (mean ± SD 4.9 ± 1.5 vs. 4.4 ± 1.5, *p* < 0.001). Treatment with NOACs was similar in both groups, while clopidogrel was more frequently used among patients allocated to mAFA intervention (14.2% vs. 7.6%, *p* = 0.011).

**Table 1:** Baseline Characteristics according to mAFA allocation and history of thromboembolism

Variables, *n* (%)	No history of thromboembolism			History of thromboembolism		
	mAFA (*n* = 1414)	Usual care (*n* = 1414)	*p*	mAFA (*n* = 232)	Usual care (*n* = 264)	*p*
Age, mean ± SD	65.6 (14.7)	69.1 (13.0)	** < 0.001**	74.5 (11.9)	75.6 (10.9)	0.295
Female sex (%)	509 (36.0)	575 (40.7)	**0.012**	116 (50.0)	62 (23.5)	** < 0.001**
Medical history						
Smokers	144 (10.2)	130 (9.2)	0.409	15 (6.5)	38 (14.4)	**0.007**
Hypertension	737 (52.1)	783 (55.4)	0.090	171 (73.7)	179 (67.8)	0.180
CAD	494 (34.9)	572 (40.5)	**0.003**	141 (60.8)	152 (57.6)	0.528
HF	226 (16.0)	301 (21.3)	** < 0.001**	134 (57.8)	53 (20.1)	** < 0.001**
Diabetes	307 (21.7)	271 (19.2)	0.103	74 (31.9)	95 (36.0)	0.388
PAD	107 (7.6)	105 (7.4)	0.943	65 (28.0)	67 (25.4)	0.574
Renal dysfunction	81 (5.7)	125 (8.8)	**0.002**	57 (24.6)	47 (17.8)	0.082
Pulmonary hypertension	49 (3.5)	56 (4.0)	0.551	38 (16.4)	27 (10.2)	0.058
Liver dysfunction	33 (2.3)	27 (1.9)	0.514	22 (9.5)	21 (8.0)	0.657
Prior brain bleeding	7 (0.5)	17 (1.2)	0.065	17 (7.3)	21 (8.0)	0.926
Prior other bleeding	30 (2.1)	39 (2.8)	0.330	24 (10.3)	28 (10.6)	1.000
Hyperthyroidism	24 (1.7)	28 (2.0)	0.675	13 (5.6)	23 (8.7)	0.247
Dilated cardiomyopathy	30 (2.1)	40 (2.8)	0.276	14 (6.0)	21 (8.0)	0.511
Hypertrophic cardiomyopathy	14 (1.0)	13 (0.9)	1.000	11 (4.7)	16 (6.1)	0.654
Anemia	38 (2.7)	60 (4.2)	**0.031**	38 (16.4)	35 (13.3)	0.394
Type of AF			** < 0.001**			0.396
Unknown	265 (18.9)	100 (7.1)		16 (7.0)	13 (4.9)	
New-onset AF	186 (13.3)	212 (15.0)		9 (3.9)	20 (7.6)	
Paroxysmal AF	586 (41.8)	556 (39.3)		87 (37.8)	104 (39.5)	
Persistent AF	303 (21.6)	373 (26.4)		77 (33.5)	75 (28.5)	
Long-standing AF	37 (2.6)	80 (5.7)		19 (8.3)	21 (8.0)	
Permanent AF	26 (1.9)	93 (6.6)		22 (9.6)	30 (11.4)	
Prior AF treatment						
Pharmacological cardioversion	177 (12.5)	127 (9.0)	**0.003**	36 (15.5)	28 (10.6)	0.135
Electrical cardioversion	24 (1.7)	19 (1.3)	0.539	6 (2.6)	16 (6.1)	0.098
AF ablation	167 (11.8)	146 (10.3)	0.231	16 (6.9)	27 (10.2)	0.248
Pacemaker	55 (3.9)	63 (4.5)	0.510	21 (9.1)	22 (8.3)	0.901
LAAO	19 (1.3)	8 (0.6)	0.053	14 (6.0)	22 (8.3)	0.417
Scores						
CHA2DS2-VASc, mean ± SD	2.46 (1.37)	2.42 (1.31)	0.487	4.89 (1.47)	4.39 (1.47)	** < 0.001**
HAS-BLED, mean ± SD	1.19 (0.92)	1.31 (0.85)	** < 0.001**	2.55 (1.16)	2.57 (1.09)	0.850

*AF* atrial fibrillation, *CAD* coronary artery disease, *CHF* congestive heart failure, *CKD* chronic kidney disease, *IQR* interquartile range, *LAAO* left atrial appendage occlusion, *PAD* peripheral artery disease, *SD* standard deviation, *TE* thromboembolic events

### Patients without previous thromboembolic event

Among patients without previous history of thromboembolism at baseline, 1414 (50%) were allocated to mAFA intervention and 1414 (50%) to usual care. Patients in the mAFA intervention group were younger (65.6 ± 14.7 vs. 69.1 ± 13.0 years, *p* < 0.001), less likely females (*p* = 0.012), and with lower prevalence of CAD, HF, renal dysfunction and anemia; they were also treated more frequently with NOAC (63.2% vs. 36.0%, *p* < 0.001) and less prescribed with warfarin or antiplatelets.

### Risk of major outcomes according to mAFA intervention

To ensure balance of baseline characteristics in the subgroups according to mAFA allocation and history of thromboembolism, we computed IPTW based on subgroup-balancing PS. Balance assessment of baseline characteristics before and after IPTW in patients with and without previous thromboembolism is reported in Supplemental Table 3 and 4, respectively. Overall, baseline characteristics were adequately balanced between mAFA intervention and usual care group in both groups of patients.

**Fig. 2:**
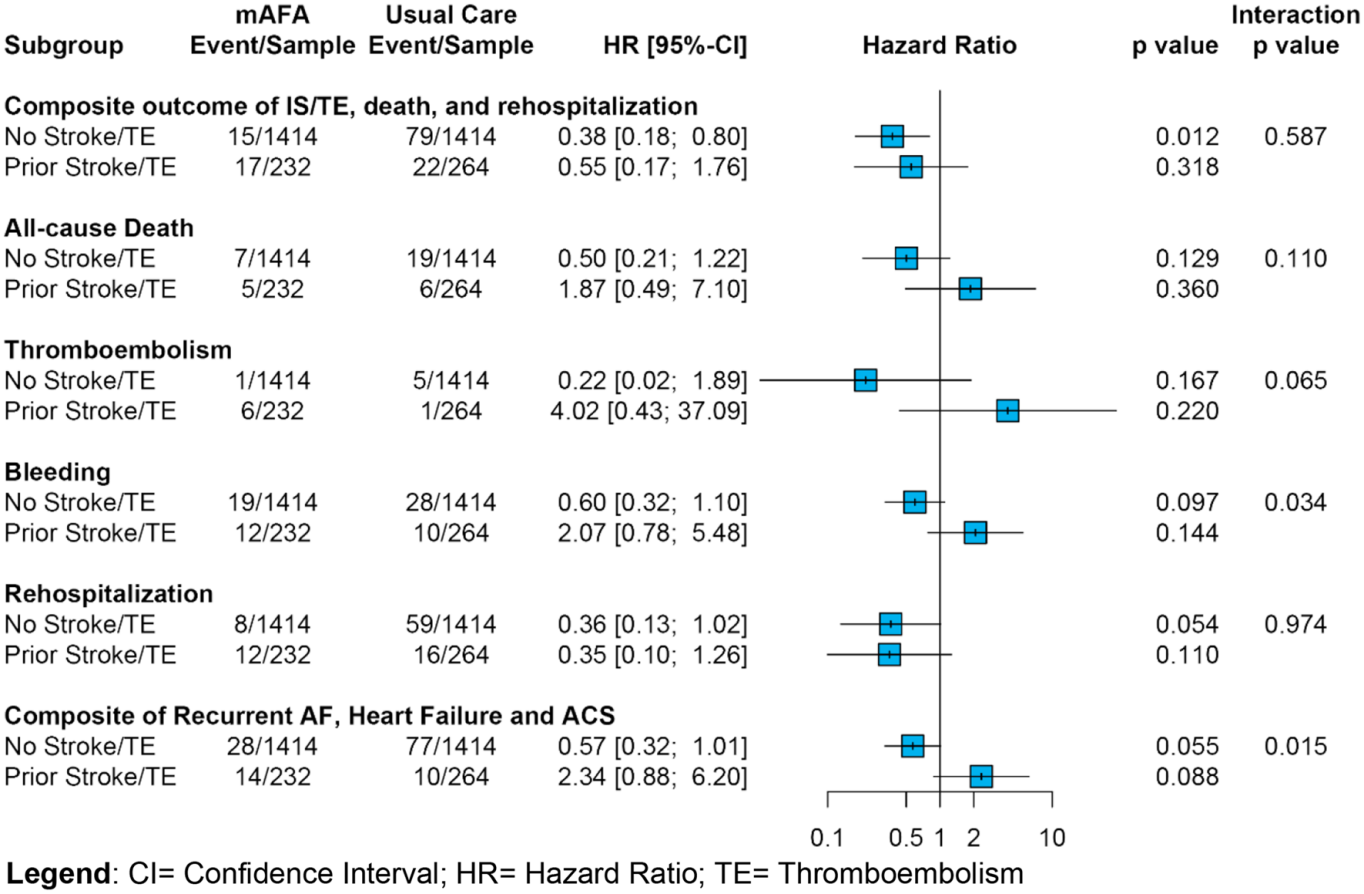
Risk of major outcomes according to mAFA intervention in patients with vs. without history of thromboembolic events. *CI* confidence interval, *HR* Hazard ratio, *IS* Ischemic stroke, *TE* thromboembolism

Results of the analysis on the association between mAFA intervention and risk of major outcomes are reported in Fig. 2 and Table 2. No statistically significant interaction was observed (*p*_int_ = 0.587) for the association between mAFA intervention and risk of the primary composite outcome of all-cause death, ischemic stroke or thromboembolism and rehospitalization among those with history of thromboembolism (HR 0.55, 95% CI 0.17–1.76) and those without (HR 0.38, 95% CI 0.18–0.80).

**Table 2:** Clinical outcomes in mAFA intervention and Usual Care groups according to history of thromboembolism

Outcome	Number of events		IR (95% CI) per 100 persons-year				
	mAFA	Usual care	mAFA	Usual care	HR (95%CI)*	*p*	interaction *p*
Composite outcome of IS/TE, death and rehospitalization							
Without history of prior TE	15/1414	79/1414	1.5 [0.8–2.4]	7.1 [5.7–8.9]	0.38 [0.18–0.80]	0.012	0.587
With history of prior TE	17/232	22/264	12.2 [8.1–19.5]	12.9 [8.1–19.5]	0.55 [0.17–1.76]	0.318	
All-cause death							
Without history of prior TE	7/1414	19/1414	0.7 [0.3–1.4]	1.7 [1.0–2.6]	0.51 [0.21–1.22]	0.129	0.110
With history of prior TE	5/232	6/264	3.4 [1.1–8.0]	3.3 [1.2–7.2]	1.87 [0.49–7.10]	0.360	
Thromboembolism (IS or systemic embolism)							
Without history of prior TE	1/1414	5/1414	0.1 [0.0–0.5]	0.4 [0.1–1.0]	0.22 [0.02–1.89]	0.167	0.065
With history of prior TE	6/232	1/264	4.1 [1.5–9.0]	0.5 [0.0–3.0]	4.02 [0.43–37.09]	0.220	
Bleeding							
Without history of prior TE	19/1414	28/1414	1.9 [1.1–2.9]	2.5 [1.6–3.6]	0.60 [0.32–1.10]	0.097	**0.034**
With history of prior TE	12/232	10/264	8.4 [4.3–14.7]	5.6 [2.7–10.2]	2.07 [0.78–5.48]	0.144	
Rehospitalization							
Without history of prior TE	8/1414	59/1414	0.8 [0.3–1.5]	5.3 [4–6.8]	0.36 [0.13–1.02]	0.054	0.974
With history of prior TE	12/232	16/264	8.5 [4.4–14.8]	9.2 [5.2–14.9]	0.35 [0.10–1.26]	0.110	
Composite of recurrent AF, heart failure and acute coronary syndrome							
Without history of prior TE	28/1414	77/1414	2.8 [1.8–4.0]	6.9 [5.4–8.6]	0.57 [0.32–1.01]	0.055	**0.015**
With history of prior TE	14/232	10/264	9.8 [5.4–16.4]	5.5 [2.7–10.2]	2.34 [0.88–6.20]	0.088	

HR [95% CI] after IPTW-Cox regressoi analysis

*AF* atrial fibrillation, *HR* hazard ratio, *IR* incidence rate, *IS* Ischemic stroke, *TE* thromboembolic event

The analysis of the exploratory secondary outcomes showed a significant interaction between history of thromboembolism and the effect of mAFA for bleeding events (*p*_int_ = 0.034) and the composite outcome of recurrent AF, heart failure and acute coronary syndromes (*p*_int_ = 0.015). Patients with previous thromboembolism allocated to mAFA intervention had non-significant trends towards more bleeding events and the composite outcome of recurrent AF, heart failure and acute coronary syndrome. Similar, non-statistically significant trends were observed for thromboembolism and mortality (Fig. 2; Table 2).

## Discussion

In this post-hoc analysis of the mAFA-II trial on secondary prevention AF patients, our main results are the following: (1) patients with history of stroke or systemic embolism had a significantly higher burden of cardiovascular risk factors and comorbidities compared to those without prior stroke or systemic embolism; (2) the effect of mAFA intervention on the primary composite outcome of all-cause death, ischemic stroke or systemic embolism, and rehospitalization remained consistent in both patients in primary or secondary thromboembolic risk prevention, with no statistically significant interaction; and (3) the effect mAFA intervention on the risk of the exploratory secondary outcomes was lower among patients with previous thromboembolism.

Although the rate of ischemic stroke in patients with AF is declining, a significant proportion of AF patients still experience thromboembolic events 17, and those with previous ischemic stroke are at particularly high risk [18]. These patients require significant efforts to improve their prognosis, beyond anticoagulation per se, using a pragmatic holistic and integrated care management perspective [14].

Indeed, our study shows that AF patients with a history of ischemic stroke or systemic embolism have a significantly higher burden of cardiovascular risk factors and comorbidities. Accordingly, previous studies showed a high prevalence of different cardiovascular and non-cardiovascular comorbidities among patients with AF and previous ischemic stroke, which significantly contributed to higher risk of future thromboembolic events [19, 20]. Indeed, this is consistent with the ‘stroke-heart syndrome’ concept, whereby there is a high risk of incident cardiovascular events in the 4 weeks post-stroke, which in turn led to higher risks of mortality, hospitalization and in some cases, recurrent stroke [17, 21]. Unsurprisingly, in our study patients with previous ischemic stroke or systemic embolism had higher rates of major adverse events, thus emphasizing how these subjects represent a group at high cardiovascular risk, and an overall ‘complex-to-treat’ population [14].

Nevertheless, the primary analysis of the mAFA-II has shown that an integrated AF treatment plan, incorporating the mHealth-implemented ABC pathway, significantly reduced the risk of the primary outcome in patients with AF [13]. In this post-hoc analysis, we show how this effect remained consistent among patients with and without history of previous stroke or systemic embolism, although attenuated in those in secondary thromboembolic risk prevention.

Our results are consistent with previous evidence on the efficacy of the ABC pathway pathway in clinically complex patients [22], and with previous post-hoc analyses from the mAFA-II trial which focused on other high-risk groups [23]. However, this analysis is the first with a specific focus on high-risk AF patients with previous thromboembolic events, and the trend towards lower effect may suggests that secondary prevention AF patients may require further specific interventions to improve their prognosis.

Indeed, several hypotheses can explain these findings. First, patients with stroke usually requires integrated and tailored approach to promote rehabilitation and achieve better functional outcomes [24], as well as better cardiovascular prognosis [25]; exercise, as well as management of psychological and social sequelae of stroke, also have a pivotal role in the prevention of sequelae and recurrent events [26–28]. Furthermore, assessment and management of frailty—which is common in both AF and stroke patients [29, 30]—may have an additive role in improving overall outcomes in stroke patients, especially given the relationship between frailty and stroke prognosis [31, 32]. This is also particularly important considering the trend observed for the risk of the exploratory secondary outcomes in secondary prevention patients allocated to mAFA intervention. All these factors may represent some specific action points in the context of an integrated care approach for post-stroke patients. Moreover, social determinants of health [33, 34], as well as socioeconomical and educational factors [35, 36], are already recognized as factors that may influence prognosis in stroke patients, and may therefore have a specific role in the secondary prevention of patients with AF. A well-designed and structured management plan is needed in order to improve the prognosis of these subjects.

Consistently, our findings suggest that an ABC-adherent management is associated with improved outcomes among AF patients, as previously shown [37, 38], but those with previous stroke or thromboembolism, given their underlying clinical complexity, may require a dedicated and integrated bundle of care to achieve an appropriate management of the increased thromboembolic risk. This hypothesis is consistent with a recent position paper, proposing the implementation of an integrated “post-stroke ABC pathway” [14], to optimize the management of these patients, based on three main pillars: (a) appropriate antithrombotic therapy, (b) better functional and psychological status; and (c) cardiovascular risk factors and comorbidities optimization, which also includes lifestyle changes. [39]

### Strengths and limitations

This is the first analysis to investigate the effect of a mHealth-implemented ABC pathway in AF patients with previous thromboembolism, thus offering an unparalleled outlook on a high-risk subgroup of AF patients. Furthermore, the results on the primary outcome were consistent with the main trial’s analysis. However, our study has also some limitations. First, this is a post-hoc analysis, and therefore lacks statistical power in the subgroup of patients with history of previous thromboembolism. This particularly apply to the results of the secondary outcomes, especially given the relative low number of events; therefore, these findings should be regarded as explorative. Despite the differences of baseline characteristics among patients allocated to mAFA intervention and usual care in both groups, we implemented an IPTW analysis using a subgroup-balancing PS based on an extensive number of variables. Notwithstanding these limitations, we cannot exclude the contribution of other unaccounted confounders on the results observed, and the results reported should be therefore interpreted with caution. Finally, we were unable to analyze data regarding socioeconomical factors or quality of anticoagulation, which may have influenced the results observed and the difference between primary and secondary prevention AF patients.

## Conclusion

In this post-hoc analysis of the mAFA-II trial, a mHealth-technology-implemented ABC pathway was associated with a reduction of the primary composite outcome of all-cause death, ischemic stroke or systemic embolism, and rehospitalization among AF patients, with consistent effect among AF patients with and without a previous history of thromboembolism. Our findings support the need for an integrated and tailored approach dedicated to stroke patients, to overcome their complexity in clinical practice and improve their prognosis.

## Data Availability

Data supporting the current study are available from the corresponding author upon reasonable request.
